# Aging enhances liver fibrotic response in mice through hampering extracellular matrix remodeling

**DOI:** 10.18632/aging.101124

**Published:** 2016-12-09

**Authors:** Bénédicte Delire, Valérie Lebrun, Charlotte Selvais, Patrick Henriet, Amélie Bertrand, Yves Horsmans, Isabelle A. Leclercq

**Affiliations:** ^1^ Laboratory of Hepato-Gastroenterology, Institut de Recherche Expérimentale et Clinique (IREC), Université catholique de Louvain (UCL), Brussels, Belgium; ^2^ Cell Biology Unit, de Duve Institute, Université catholique de Louvain, Brussels, Belgium; ^3^ Department of Hepato-Gastroenterology, Cliniques Universitaires Saint-Luc and Institute of Clinical Research, Université catholique de Louvain, Brussels, Belgium

**Keywords:** macrophages, collagen crosslinking, fibrosis resolution, matrix metalloproteinase, inflammation

## Abstract

Clinical data identify age as a factor for severe liver fibrosis. We evaluate whether and how aging modulates the fibrotic response in a mouse model. Liver fibrosis was induced by CCl_4_ injections (thrice weekly for 2 weeks) in 7 weeks- and 15 months-old mice (young and old, respectively). Livers were analyzed for fibrosis, inflammation and remodeling 48 and 96 hours after the last injection. Old mice developed more severe fibrosis compared to young ones as evaluated by sirius red morphometry. Expression of pro-fibrogenic genes was equally induced in the two age-groups but enhanced fibrolysis in young mice was demonstrated by a significantly higher *Mmp13* induction and collagenase activity. While fibrosis resolution occurred in young mice within 96 hours, no significant fibrosis attenuation was observed in old mice. Although recruitment of monocytes-derived macrophages was similar in young and old livers, young macrophages had globally a remodeling phenotype while old ones, a pro-fibrogenic phenotype. Moreover, we observed a higher proportion of thick fibers and enhanced expression of enzymes involved in collagen maturation in old mice.

**Conclusion:**

Impaired fibrolysis of a matrix less prone to remodeling associated with a pro-inflammatory phenotype of infiltrated macrophages contribute to a more severe fibrosis in old mice.

## INTRODUCTION

Liver fibrosis results from a sustained wound healing response due to chronic liver injury and occurs when extracellular matrix (ECM) production exceeds ECM degradation. Activated hepatic stellate cells (aHSCs) are the main cells involved in fibrogenesis as the key source of ECM compounds and a major modulator of hepatic inflammation. Next to aHSCs, the hepatic macrophages also promote fibrosis progression by driving HSCs activation, by releasing pro-inflammatory and pro-fibrogenic factors and by supporting the infiltration of pro-fibrogenic immune cells [1].

Liver fibrosis reversibility has been documented for several years. In animal models, liver damages reverse and fibrotic scar degradation occurs when the hepatotoxic agent is removed or when a normal biliary outflow is restored after common bile duct ligation [2,3]. Evidences of fibrosis regression come also from clinical practice, especially after the arrival of new anti-viral therapies enabling high rate of hepatitis C virus (HCV) eradication [4,5]. During fibrosis resolution, aHSCs disappear by senescence, inactivation or apoptosis [6–8] while inflammatory and pro-fibrogenic macrophages differentiate into pro-resolution cells able to secrete large quantities of fibrolytic matrix metallo-proteinases (MMP) and anti-inflammatory cytokines [9;10]. Thick and paucicellular fibrotic septae, collagen cross-linking and reduced production and/or activity of MMPs render the fibrotic liver less amenable to remodeling and repair [11].

The human liver is affected by aging. It manifests by a reduced volume and blood flow as well as by cellular changes such as increased oxidative stress, decreased number and dysfunction of mitochondria, accelerated cellular senescence and decreased regenerative ability [12]. Aging is also a risk factor for several specific hepatic diseases. In non-alcoholic fatty liver disease (NAFLD), evolution from simple steatosis to steato-hepatitis and fibrosis occurs more frequently in old patients [13]. In HCV chronic infection, age at time of infection is a strong determinant of fibrosis progression while liver graft from older donors is associated with a more rapid progression of HCV-related cirrhosis in the recipient [14,15]. Although those data emphasize the susceptibility due to aging to develop more severe disease and significant fibrosis, the mechanisms underlying this propensity are not fully understood. In viral hepatitis, an impaired immune response against foreign antigens may explain a different immuno-pathogenesis in the elderly and more sustained hepatic fibrotic process [16]. In rodents, a more severe fibrosis is also observed in older animals but mechanisms remain debated. Aging-dependent hepatic susceptibility to toxic agents, reduced ECM proteins degradation and variation in inflammatory cells infiltrating the injured liver are discussed [17–19] as differences in rodent genetic strains may explain at least partially divergent results. Interestingly, Karsdal et al measured specific fragments of selected ECM proteins in the serum of normal animals and concluded to a quantitatively different ECM turnover according to the age of rats. Indeed, type I and II collagen turnover was significantly reduced in old compared to young animals, while type IV and V collagen and biglycan degradation biomarkers were significantly upregulated in old rats [20].

In this work, as previously described in other studies [17,18], we reproduced a higher susceptibility to fibrosis in old mice compared to young mice after repetitive administrations of carbon tetrachloride (CCl_4_). We provide explanations for mechanisms contributing to age-related fibrosis.

## RESULTS

### Liver fibrosis is more severe in old mice independently of profibrogenic processes

**Figure 1:**
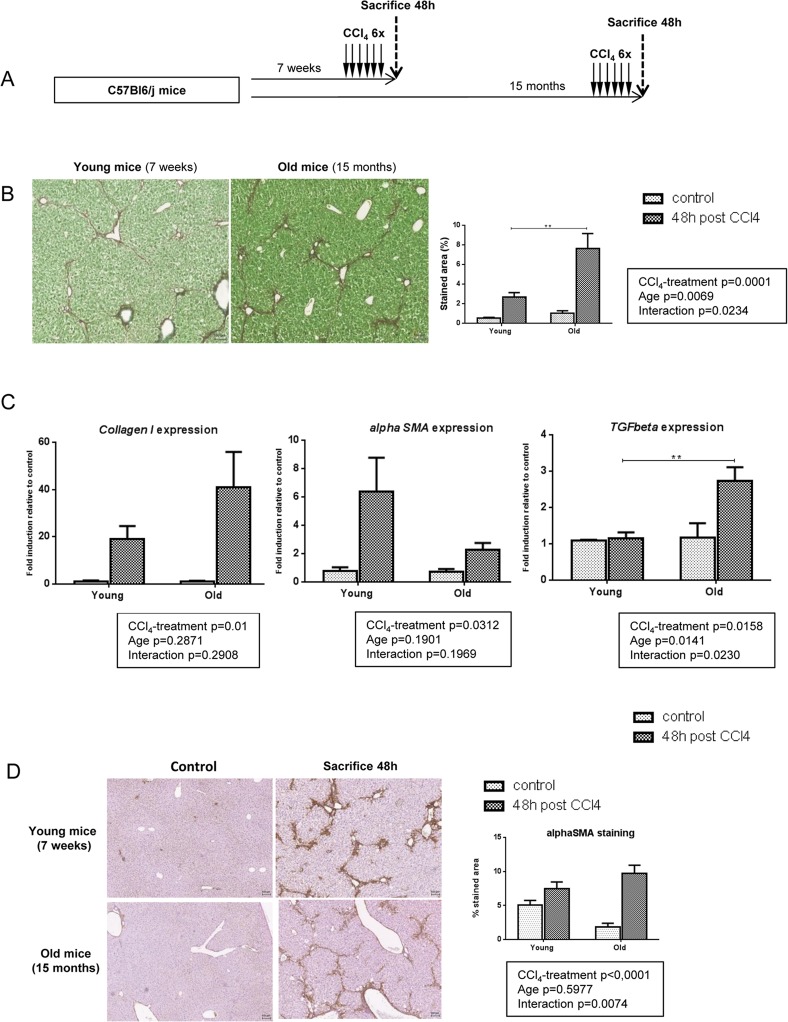
More severe liver fibrosis in old mice independently of profibrogenic processes (**A**) CCl_4_ was injected three times a week for two weeks to young and old mice (n=6/group). Livers were harvested two days after the last injection. (**B**) Sirius red stained liver sections in CCl_4_-treated mice (magnification 80x). Scale bare 100μm. Collagen fibers were evaluated as percentage of stained area in the section (n=6/group). (**C**) Hepatic gene expression of *Collagen I*, *alphaSma* and *Tgfbeta* (Mean ± SEM) (n=6/group). (**D**) Activated stellate cells were identified by alphaSMA immunohistochemistry staining in young and old mice 48 hours after the last CCl_4_ injection (magnification 80x) (n=6/group). Scale bare 100μm. Statistical analysis was performed by two-way ANOVA for repeated measures (boxes) followed by Bonferroni's post-hoc correction. **P<0.01 for differences between age groups.

No difference was observed in terms of body weight, liver to body weight ratio and hepatic macroscopic and microscopic aspect between young (aged 7 weeks) and old (aged 15 months) mice (data not shown). Fibrosis was induced by CCl_4_ injections (3x/week for 2 weeks) in young and old mice (Figure 1A). Liver damages were evaluated two days after the last CCl_4_ injection, a time point known to correspond to the peak of fibrosis [21].

In response to CCl_4_, fibrosis was significant in both age-groups compared to matched control animals but old mice developed more severe fibrosis compared to young ones as evaluated by sirius red quantification (Figure 1B). While *Transforming growth factor beta* (*Tgfbeta*) mRNA was more expressed in the old group compared to the young one, the expression of the main genes related to pro-fibrogenic processes such as *Collagen I* and *alpha Smooth muscle actin (alphaSMA)* was equally induced by CCl_4_ injections (Figure 1C). Moreover, alphaSMA positive cells were similarly distributed around portal area and fibrotic bands in both age-groups (Figure 1D). These results suggest that the more severe fibrotic scar observed in old mice was not due to enhanced matrix deposition.

### Impaired fibrolysis precludes fibrosis reversal in old mice

**Figure 2:**
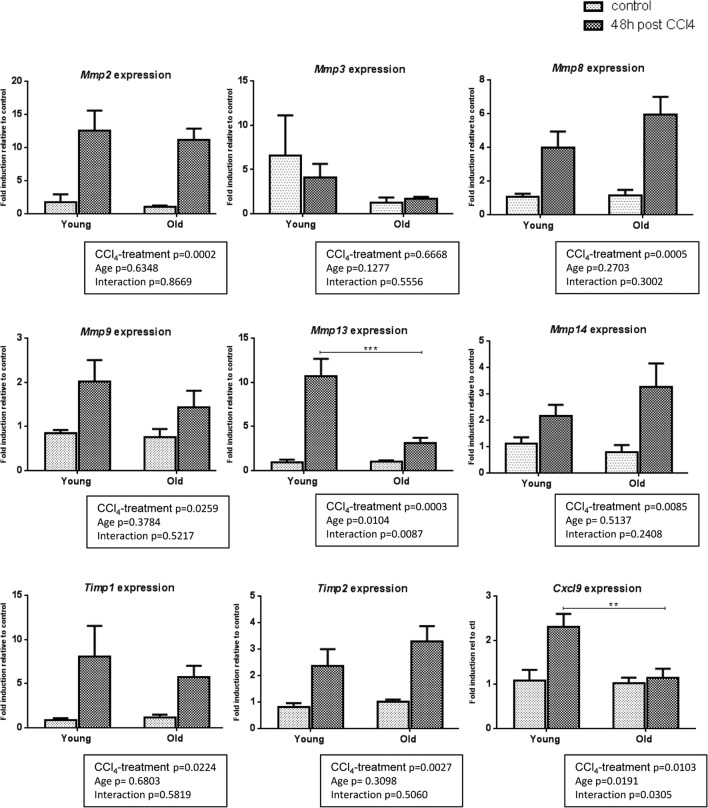
Gene expression of matrix remodeling enzymes in young and old livers Hepatic gene expression of *Mmp2*, *Mmp3*, *Mmp8*, *Mmp9*, *Mmp13*, *Mmp14*, *Timp1*, *Timp2 a*nd *Cxcl9* in control and CCl_4_-treated young and old mice (Mean ± SEM) (n=6/group). Statistical analysis was performed by two-way ANOVA for repeated measures (boxes) followed by Bonferroni's post-hoc correction. **P<0.01; ***P<0.001 for differences between age groups.

Liver fibrosis is a dynamic process resulting from an imbalance between ECM production (fibrogenesis) and degradation (fibrolysis). As we showed that fibrogenic processes were unlikely to account for the difference in intensity of fibrosis observed between groups, we studied the gene expression of matrix remodeling enzymes. CCl_4_ induced similarly *Mmp2*, *Mmp3*, *Mmp8*, *Mmp9, Mmp14* and MMP inhibitors *Tissue inhibitor metalloproteinase 1* (*Timp1)* and *Timp2* in the two age-groups but we observed a significantly higher induction of *Mmp13* in young mice than in old ones. In addition, *Chemokine (C-X-C motif) ligand 9* (*Cxcl9),* a potent MMP-13 inducer, is upregulated in young fibrotic livers but not in old ones (Figure 2).

**Figure 3:**
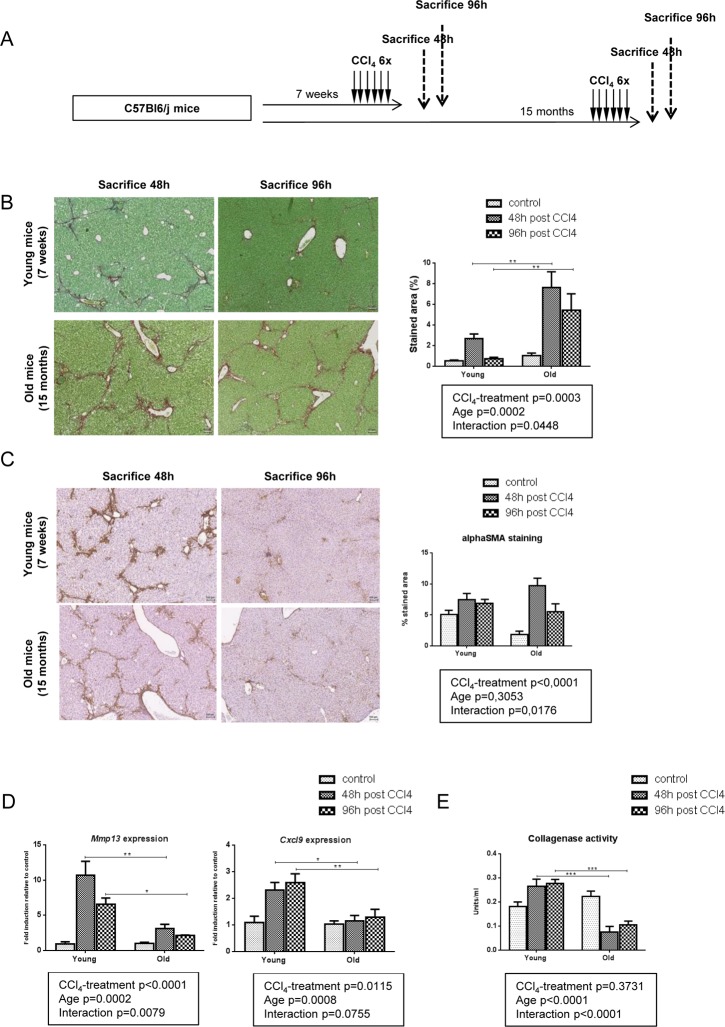
Impaired fibrolysis precludes fibrosis reversal in old mice (**A**) CCl_4_ was injected three times a week for two weeks to young and old mice (n=6/group). Livers were harvested 48h or 96h after the last injection. (**B**) Sirius red stained liver sections in CCl_4_-treated young and old mice (magnification 80x). Collagen fibers were evaluated as percentage of stained area in the section (n=6/group). Scale bare 100μm. (**C**) Activated stellate cells were identified by alphaSMA immunohistochemistry staining in young and old mice 48 and 96 hours after the last CCl_4_ injection (magnification 80x) (n=6/group). Scale bare 100μm. (**D**) Hepatic gene expression of *Mmp13* and *Cxcl9* (Mean ± SEM) (n=6/group). (**E**) Collagenase activity was measured in controls and CCl_4_-treated groups (Mean ± SEM) (n=6/group). Results are expressed in units/ml. One unit of collagenase activity is defined as the cleavage of 1 mg of collagen per minute. Statistical analysis was performed by two-way ANOVA for repeated measures (boxes) followed by Bonferroni's post-hoc correction. *P<0.05; **P<0.01 for differences between age groups.

MMP-13 is a matrix metalloproteinase chiefly involved in fibrosis regression, acting against numerous ECM components, and in particular fibrillar collagens [22]. The lower expression of this enzyme in old mice supports a less dynamic matrix remodeling in this group and could thereby contribute to a reduced fibrosis clearance. To confirm this hypothesis, we repeated the same experiment but mice were sacrified 4 days after the last injection leaving two extradays for fibrosis resolution (Figure 3A). At this time point and compared with liver harvested at peak of fibrosis (48h post last CCl_4_ injection), remodeling was significant in young mice with only some residual collagen deposition. By contrast, there was no significant attenuation of liver fibrosis between the 48- and the 96-hour time points in old mice (Figure 3B). In both groups, alphaSMA positive cells started to redistribute through the lobule 4 days after the last injection (Figure 3C). These results confirmed the impaired fibrolysis in old mice. Moreover, *Mmp*13 and *Cxcl9* gene expression levels remained low at all times while being strongly induced in young mice (Figure 3D). We then performed collagenase assay on liver homogenates in all age-groups to evaluate the ability of our samples to cleave collagen, the main substrate of MMP-13. We observed a significantly reduced collagenase activity in old mice 48 and 96 hours after the last injection of CCl_4_ (Figure 3E). These results demonstrated that a low stimulation of the CXCL9-MMP-13 axis associated with a reduced collagenolytic activity and impaired fibrosis remodeling in old mice.

### Pro-resolutive macrophages infiltrate young livers and support a more dynamic matrix remodeling compared to old mice

**Figure 4:**
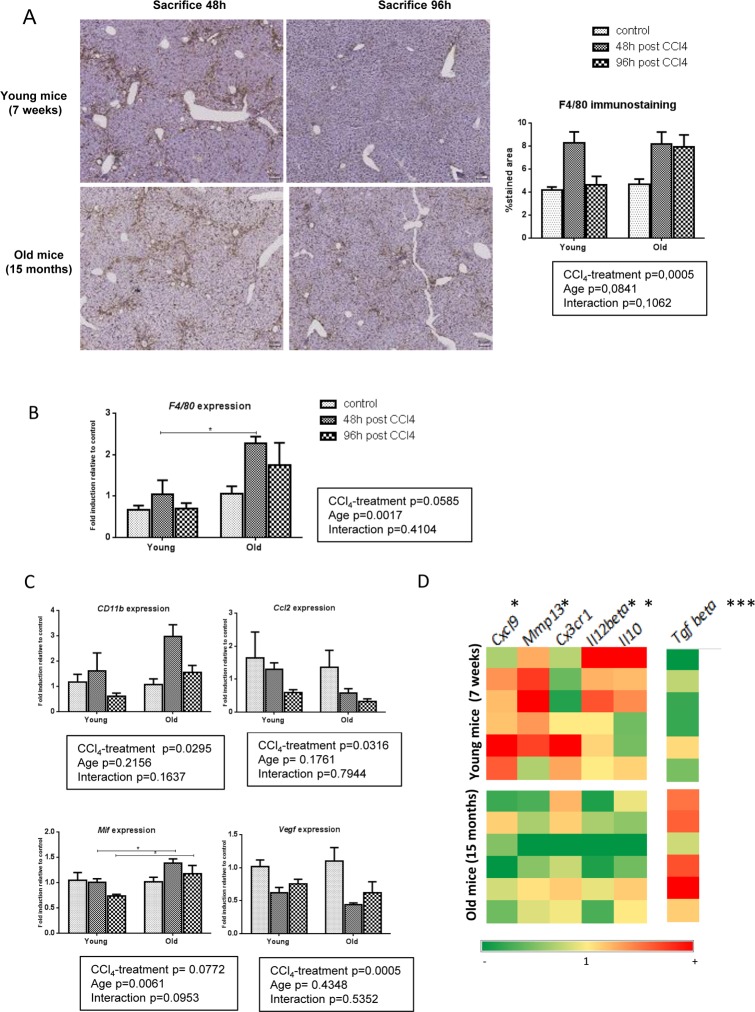
Pro-resolutive macrophages infiltrate young livers (**A**) Kupffer cells were identified by F4/80 immunohistochemistry staining in young and old mice 48 and 96 hours after the last CCl_4_ injection (magnification 80x) (n=6/group). Scale bare 100μm. Hepatic gene expression of *F4/80* (**B**) and, *CD11b*, *Ccl2*, *Mif* and Vegf (**C**) (Mean ± SEM) (n=6/group). (**D**) Hepatic genes expression of *Mmp13*, *Cxcl9*, *Cx3cr1*, *Il12beta, Il10* and *Tgf beta* (n=6/group). Induction compared to untreated liver appears in red and repression in green. Statistical analysis was performed by two-way ANOVA for repeated measures (boxes) followed by Bonferroni's post-hoc correction. *P<0.05; ***P<0.001 for differences between age groups.

CXCL9 is produced by macrophages and stimulates macrophages in an autocrine or paracrine manner to produce MMP-13 [9,23]. We used F4/80 immunohisto-chemistry and gene expression to assess liver macrophages infiltration. In young mice, F4/80 positive cells agglomerated in pericentral area and fibrotic bands at peak fibrosis (48h) and homogenously redistributed through the entire lobule upon fibrosis resolution (96h). By contrast, in old mice, enlarged macrophages persisted in the central area and around the fibrotic septa at 96h post last CCl_4_ doses (Figure 4A). Moreover, *F4/80* gene expression was higher in the liver of old mice compared to young mice (Figure 4B). This suggests distinct macrophages populations and activities in relation to aging. Macrophages involved in liver fibrosis are mainly derived from circulating monocytes. Initially pro-inflammatory pro-fibrogenic cells, they switch then to a tissue restorative phenotype to participate in ECM clearance [10,24]. We evaluated the expression of *CD11b*, a marker of freshly infiltrating monocyte-derived macrophages, as well as the expression of *Chemokine (C-C motif) ligand 2* (*Ccl2*), *Vascular endothelial growth factor* (*Vegf*) and *Macrophages migration inhibitor factor* (*Mif*), all soluble factors involved in tissue recruitment of monocytes [25]. While *Mif* was significantly more expressed in old mice compared to young ones, no difference of *Ccl2*, *Vegf* or *CD11b* gene expression was observed between age-groups suggesting similar inflammatory cells recruitment in young and old livers (Figure 4C). We then asked the question: is the liver macrophages phenotype different between young and old mice following CCl_4_-induced injury? We used the M1/M2 dichotomy and observed a M1 phenotype in young mice while old mice expressed more M2 markers (Supplementary Figure 1). We then checked the expression of both pro-fibrogenic and pro-resolutive macro-phages markers in both groups at peak of fibrosis compared to untreated controls. In the young group, macrophages had globally a higher expression of resolutive genes, especially *Mmp13* and *Cxcl9* as described above, while old mice have prominent liver expression of *Tgfbeta*, a major pro-fibrogenic marker (Figure 4D).

### Collagen cross-linking limits reversibility of liver fibrosis in old mice

**Figure 5:**
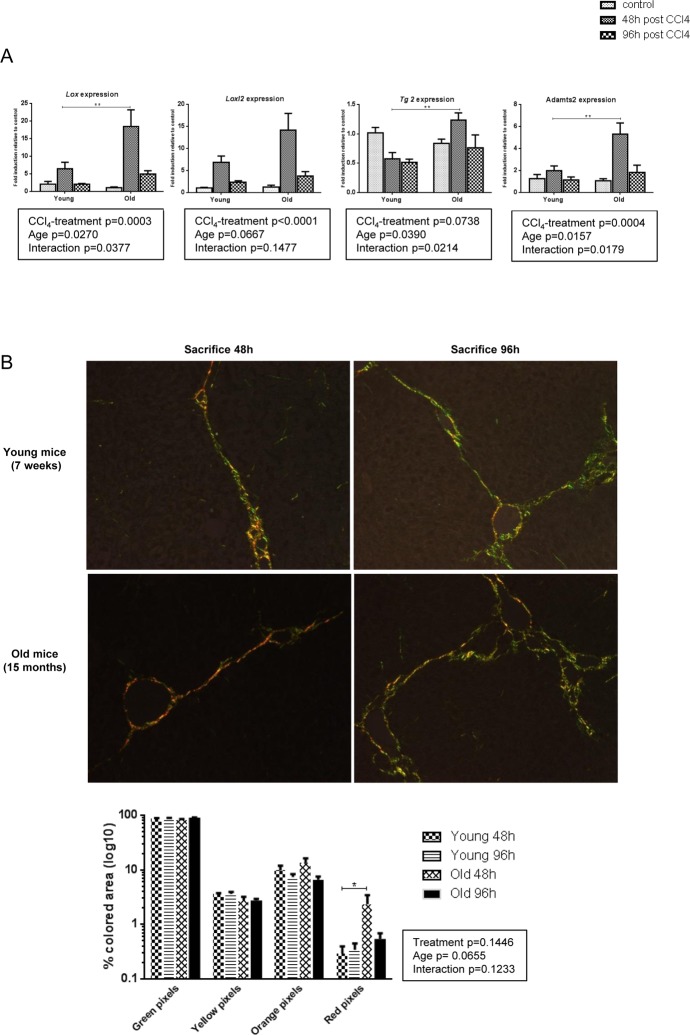
More pronounced collagen cross-linking process in old mice (**A**) Hepatic genes expression of *Lox*, *Loxl2, Tg2* and *Adamts2* (Mean ± SEM) (n=6/group). (**B**) Sirius red stained sections were observed under polarized light microscopy (200x magnification) to evaluate fiber thickness according to their color. The four different colored collagen fibers were quantified by morphometrical analysis (see methods) (n=6/group). Statistical analysis was performed by two-way ANOVA for repeated measures (boxes) followed by Bonferroni's post-hoc correction. *P<0.05; **P<0.01 for differences between age groups.

Post-translational processing of pro-collagen molecules and covalent cross-linking of collagen fibrils participates in the development of a mature and stabilized ECM and is an important limitation factor of fibrosis reversibility. Lysyl oxidase (LOX), LOX like 2 (LOXL2), a disintegrin and metalloproteinase with thrombospondin type I motif (ADAMTS2) and, while more controversial, tissue transglutaminase 2 (TG2) mediate ECM stabilization [26–29]. We evaluated the mRNA expression of these enzymes in our groups. In young mice, CCl_4_ caused a modest upregulation of *Lox* and *Loxl2* while levels of *Tg2* and *Adamts2* were unchanged (Figure 5A). By contrast, expression of *Lox, Tg2,* and *Adamts2* was significantly induced in old animals at time of peak fibrosis suggesting that in the aged liver cross-linking of the matrix proteins was more prominent. Moreover, the proportion of thick and dense red collagen fibers as visualized under polarized light was significantly higher in old mice at peak of fibrosis (Figure 5B). More pronounced cross-linking process in old mice would promote excess accumulation of thicker fibrotic septa and confer resistance to ECM proteolytic degradation even after the cessation of liver injury.

## DISCUSSION

The worldwide increasing proportion of elderly people leads to an increasing incidence of some hepatic disorders and complications for which age is a risk factor, among them fibrosis. Moreover, liver disease related treatments are more and more frequently proposed to elderly people, such as HCV-infected patients. Therefore, the impact of aging on liver fibrosis, the main adverse effect of chronic liver disorders, is of major interest.

In this animal study, we applied the same profibrotic regimen to young and old mice and we observed a more severe fibrosis in old mice compared to young ones at the peak of fibrosis. These results are in concordance first with clinical observations suggesting that susceptibility to fibrosis increases with age in many liver diseases [13–15] and, second with animal studies. Indeed, several works describe a more severe fibrosis in old animals (mice and rats) after repeated exposure to CCl_4_ [17,18].

A single dose of CCl_4_ disrupts centrilobular hepatocytes integrity that wound healing processes tend to restore. In case of repeated exposures, recurrent profibrotic stimulation occurs prior to the resolution of the previous healing round [30]. In our study, *Collagen I* and *alphaSma* mRNA were significantly upregulated in treated groups compared to controls but no difference was observed between age-groups, mitigating the role of variable fibrogenic processes in the severity of ECM deposition. Rather, this is in favor of an equal propensity to initiate profibrotic events in response to a toxic injury. Moreover, here, as in previous works [31], acute CCl_4_-induced hepatotoxicity was similar in young and old animals suggesting that CCl_4_ was transformed in toxic species in a same way independently of the age.

Long time described as a static and irreversible phenomenon, fibrotic scar deposition is now recognized as a dynamic and bidirectional process emphasizing the role of ECM remodeling enzymes [4]. MMPs are zinc-dependent endopeptidases forming a family of over 20 enzymes. They are involved both in fibrosis progression and resolution through their ability to degrade virtually all compounds of the ECM [32,33]. The capacity of the liver to resorb scar or in the contrary to “preserve” the pathologic matrix accumulated after injury will depend on the balance between MMPs and their respective inhibitors. Among all MMPs, MMP-13 is the main interstitial collagenase in rodents and largely involved in fibrosis resolution [9,23,34,35]. We observed a strong induction of *Mmp13* gene expression in young mice at peak of fibrosis while old mice expressed significantly less *Mmp13* mRNA. No difference was noticed concerning *TIMPs* expression suggesting that the balance MMP/TIMP was overtly tilted in favor of matrix degradation in young mice but less so in old ones. This was confirmed by the nearly complete clearance of scar matrix in young animals 4 days after the last toxic injection while virtually no remodeling occurred in old mice, and by the reduced collagenolytic activity in this last group. In addition, CXCL9, a canonical inducer of MMP-13 [23], was also reduced in aged fibrotic liver.

Fibrosis and inflammation are closely linked and immune cells are strongly involved in all steps of the fibrotic process from the inflammatory response following initial injury to fibrosis regression [36]. At steady state, the bulk of hepatic macrophages derive from resident Kupffer cells while monocyte-derived recruited cells are in minority. In chronic hepatic injury, bone marrow-derived immune cells are intensively recruited by Kupffer cells in a CCL2/CCR2- and MIF-dependent manner [37–39] and VEGF regulates sinusoidal permeability to facilitate monocytes infiltration in the liver [23]. Those recruited macro-phages are increasingly seen as major contributors of the response to hepatic damages, through the release of a wide range of soluble factors driving HSCs activation and ECM synthesis, as well as having a key role in tissue architecture restoration through MMPs production [10,24,35]. Ramachandran and colleagues demonstrated that restorative macrophages derive from recruited pro-fibrotic macrophages thereby indicating a phenotypic switch [10]. These two different macro-phages populations were initially classified according to the simple dichotomous M1/M2 nomenclature. With time, it has become evident that in liver diseases, it was difficult to assign hepatic macrophages to the M1 activated group or to the M2 alternatively activated group and that a functional classification was more adequate. Pro-fibrotic macrophages support HSCs activation and ECM synthesis through the release of TGFbeta while pro-resolutive macrophages are identified by the expression of matrix-degrading enzymes [9], chemokines such as CXCL9 that stimulates macrophages to produce MMP-13 and reduces stellate cells activation [40,41] and other factors such as CX3CR1, IL10 and IL12β [42–44]. Collectively, our results suggested that macrophages recruitment was quantitatively similar in young and old mice at peak fibrosis but that an impaired phenotypic and functional switch may contribute to reduced fibrolysis and persistent fibrosis. In addition, parenchymal re-localisation of macrophages was also impaired in old mice, perhaps due to the maintenance of a scarring environment. Interestingly, several works report variable macrophages activity in relation to age. Hilmer et al. described an increased basal phagocytic activity in Kupffer cells while Yang and colleagues reported enhanced inflammatory cytokine secretion and mesenchymal stem cells attraction by old primary Kupffer cells compared to young ones [45,46]. Dynamic changes in the monocytes/macrophages lineage occur during ageing in humans also [47]. Whether the nature of the injurious stimulus for fibrosis (toxic like here, cholestatic or chronic inflammation) may differentially influence the age-associated plasticity of macrophages has not yet been investigated.

Next to the variable inflammatory response, we demonstrated a higher proportion of thick and dense fibers in old mice as well as an enhanced expression of the enzymes involved in collagen maturation changes. Septal features limit fibrosis remodeling: old, pauci-cellular, thick and heavily cross-linked septae resist to proteases degradation [11]. ADAMTS2, LOX, LOXL2 and TG2 activity mainly contributes to fibers maturation by processing immature collagen precursors and by mediating the irreversible cross-link of collagen and elastin fibers [26–29]. Data are quite controversial for TG2 as *Tg2* deletion in mice does not promote fibrosis reversal in a model of advanced liver fibrosis [48]. More than biochemical impact on matrix fibrils, cross-linking enzymes support also HSCs activation by maintaining a stiff environment and may have immuno-modulatory functions in liver fibrosis influencing the changes in balance between fibrogenesis and fibrolysis [28]. As they are mainly expressed by HSCs, one may ask the question of the impact of aging on HSCs function/reactivity in case of chronic liver injury. HSC hyperplasia is observed in the normal liver of old mice and rats, and old human HSCs have reduced telomere length [49;50] but no data exist concerning the activity of old HSC compared to young ones.

To date, no antifibrotic therapy exists besides the suppression of the causative agent. Our work, demonstrating that liver fibrosis is less prone to reverse in old animals, has several clinical implications. First, as already pointed by Karsdal et al [20], impact of aging on reduced ability for fibrosis degradation may partially explain some disappointing results of antifibrotic agents in clinical trials while promising when preclinically tested [51]. Indeed, pre-clinical studies usually use young animals (6-8 weeks old) while patients concerned by treatment classically suffer from fibrosis that has developed over decades rather than weeks in animals. Secondly, our study highlights the importance to target the correct underlying processes in the perspective of an effective therapy. Based on our results, this target may be different according to the age of the patients, and therapies supporting the fibrolysis or opposing the cross-linking of the matrix might be of particular interest in an old population.

## MATERIALS AND METHODS

### Animal model and sample preparation

Male 7-weeks-old (the young mice) and 15-months-old (the old mice) C57BL/6J mice were purchased from Elevage Janvier, Le-Genest-St-Ile, France. They were exposed to a 12h light/12h dark cycle, were maintained at a constant temperature of 20°C–22°C and received food and drink ad libitum. Young and old animals were maintained in parallel in the same facility for a total duration of one month until sacrifice. Animal care was provided in accordance to the guidelines for humane care for laboratory animals established by the Université catholique de Louvain in accordance with European regulations and in conformity with ARRIVE guidelines. The study protocol was approved by the university ethics committee (2012UCLMD026).

A fibrotic regimen (CCl_4_ intra-peritoneal injection 750μl/kg body weight of CCl_4_ diluted in corn oil, 3 times a week for 2 weeks) was applied to young and old mice and started one week after their arrival in the facility. Control animals received injections of the same volume of corn oil (n=6 in the young group and n=6 in the old group). Mice were sacrificed 48 hours (n=6 in the young group and n=6 in the old group) or 96 hours (n=6 in the young group and n=6 in the old group) after the last injection. At the time of sacrifice, mice where anesthetized and the blood was drawn by cardiac puncture. The liver was rapidly dissected out. A part of the liver was immediately immersed in 4% formalin for histological analyses and the rest snap-frozen in liquid nitrogen and kept at −80°C until analyses.

### Histology

Sirius red staining was performed on formalin-fixed and paraffin-embedded tissue. Liver fibrosis quantification (percentage of stained area in the section) was performed using Tissue IA software (Leica Biosystems, Dublin, Ireland) after digitalization with a SCN400 slide scanner (Leica Biosystems, Wetzlar, Germany). The same stained sections were then observed under polarized light microscopy to evaluate fiber thickness according to their color as previously described: thin fibers range from green to yellow while thick fibers range from orange to red [52–54]. The four different colored collagen fibers were quantified by morpho-metrical analysis using FRamework for Image Dataset Analysis (FRIDA) software (bui3.win.ad.jhu/frida/) on 5 images focused on a fibrotic septae per liver section (20xmagnification). The proportion of each color was expressed as the percentage of all colored pixels. The percentage of stained pixels for each color in each image was then averaged to give a mean score for each liver section.

Kupffer cells were identified by F4/80 immunostaining using a primary rat anti-mouse F4/80 monoclonal Ab (1:200; AbD Serotec, MCA497G, Clone A3-1; Oxford, UK), a rabbit anti-rat immunoglobulin (1:100; Vector Laboratories, AI-4001, Burlingame, USA), and then a goat anti-rabbit streptavidin horseradish peroxidase-conjugated Ab (En Vision K4003; Dako). Peroxidase activity was revealed with diaminobenzidine (DAB) and slides counterstained with hematoxylin. Activated stellate cells were identified by alphaSMA immuno-staining using a primary mouse anti-mouse alphaSMA monoclonal Ab (1:200; Dako, M0851; Santa Clara, USA) and a goat anti-mouse streptavidin horseradish peroxidase-conjugated Ab (En Vision K4001; Dako). Peroxidase activity was revealed with diaminobenzidine (DAB) and slides counterstained with hematoxylin. Immunostaining quantification (percentage of stained area in the section) was performed using Tissue IA software (Leica Biosystems, Dublin, Ireland) after digitalization with a SCN400 slide scanner (Leica Biosystems, Wetzlar, Germany).

### RNA extraction, reverse transcription, RT-qPCR

Total RNA was extracted from frozen liver samples using TRIzol Isolation Reagent (Life Technologies, Belgium). cDNA was synthesized from 1μg RNA using High-Capacity cDNA Reverse Transcription Kit (Applied Biosystems, Lennik, Belgium). Real time PCR analysis was performed in duplicate with the StepOnePlus real-time PCR System (Applied Bio-systems, Lennik, Belgium) using SYBRgreen. Primer pairs for transcripts of interest were designed using primer express design software (Applied Biosystems, Lennik, Belgium) and are listed in Supplementary Table 1. RPL19 mRNA was chosen as an invariant standard. Results are expressed as fold expression relative to expression in the control group using the ΔΔCt method.

### Collagenase assay

100 mg of frozen liver tissue samples were homogenized in ice-cold lysis buffer (50 mM HEPES, 150 mM NaCl, 1.5 mM MgCl2, 10% glycerol, 0.1% Triton X-100, 1 mM DTT, 1 mM NaF, 1 mM PMSF, 0.1 mM Na3VO4, 2 μg/ml aprotinin, 100 μg/ml leupeptin). The homogenates were centrifuged at 10,000 × *g* for 5 min at 4°C and the supernatant was stored at −80°C.

Collagenase activity of 100μl of liver homogenate was evaluated using FITC-labeled telopeptide-free soluble bovine type I collagen (Collagenase Assay Kit; Chondrex Inc, Catalog 3001, Redmond, USA) and following the manufacturer instructions.

### Statistical analysis

All the data are presented as means ± standard error of the mean (SEM). Statistical analysis was performed by two-way ANOVA for repeated measures followed by Bonferroni's post-hoc correction. Statistical significance was assumed for p values <0.05 (*p<0.05; **p<0.01; ***p<0.001). GraphPad Prism software (San Diego, CA, USA) was used for graphs and statistics.

## SUPPLEMENTARY MATERIAL



## Abbreviations

ECM: extracellular matrix
(a)HSC: (activated) hepatic stellate cell
HCV: hepatitis C virus
MMP: matrix metalloproteinase
NAFLD: non-alcoholic fatty liver disease
CCl4: carbon tetrachloride
FRIDA: FRamework for Image Dataset Analysis
SEM: standard error of the mean
TGFbeta: transforming growth factor beta
alphaSMA: alpha smooth muscle actin
TIMP: tissue inhibitor of metaloproteinase
CXCL9: chemokine (C-X-C motif) ligand 9
CCL2: chemokine (C-C motif) ligand 2
VEGF: vascular endothelial growth factor
MIF: macrophage migration inhibitor factor
LOX: lysyl oxidase
LOXL2: LOX like 2
TG2: tissue transglutaminase 2
ADAMTS2: a disintegrin and metalloproteinase with thrombospondin type I motif

## References

[1] Hernandez-Gea V, Friedman SL (2011). Pathogenesis of liver fibrosis. Annu Rev Pathol.

[2] Iredale JP, Benyon RC, Pickering J, McCullen M, Northrop M, Pawley S, Hovell C, Arthur MJ (1998). Mechanisms of spontaneous resolution of rat liver fibrosis. Hepatic stellate cell apoptosis and reduced hepatic expression of metalloproteinase inhibitors. J Clin Invest.

[3] Issa R, Williams E, Trim N, Kendall T, Arthur MJ, Reichen J, Benyon RC, Iredale JP (2001). Apoptosis of hepatic stellate cells: involvement in resolution of biliary fibrosis and regulation by soluble growth factors. Gut.

[4] Ellis EL, Mann DA (2012). Clinical evidence for the regression of liver fibrosis. J Hepatol.

[5] Hammel P, Couvelard A, O'Toole D, Ratouis A, Sauvanet A, Fléjou JF, Degott C, Belghiti J, Bernades P, Valla D, Ruszniewski P, Lévy P (2001). Regression of liver fibrosis after biliary drainage in patients with chronic pancreatitis and stenosis of the common bile duct. N Engl J Med.

[6] Kisseleva T, Cong M, Paik Y, Scholten D, Jiang C, Benner C, Iwaisako K, Moore-Morris T, Scott B, Tsukamoto H, Evans SM, Dillmann W, Glass CK, Brenner DA (2012). Myofibroblasts revert to an inactive phenotype during regression of liver fibrosis. Proc Natl Acad Sci USA.

[7] Krizhanovsky V, Yon M, Dickins RA, Hearn S, Simon J, Miething C, Yee H, Zender L, Lowe SW (2008). Senescence of activated stellate cells limits liver fibrosis. Cell.

[8] Troeger JS, Mederacke I, Gwak GY, Dapito DH, Mu X, Hsu CC, Pradere JP, Friedman RA, Schwabe RF (2012). Deactivation of hepatic stellate cells during liver fibrosis resolution in mice. Gastroenterology.

[9] Fallowfield JA, Mizuno M, Kendall TJ, Constandinou CM, Benyon RC, Duffield JS, Iredale JP (2007). Scar-associated macrophages are a major source of hepatic matrix metalloproteinase-13 and facilitate the resolution of murine hepatic fibrosis. J Immunol.

[10] Ramachandran P, Pellicoro A, Vernon MA, Boulter L, Aucott RL, Ali A, Hartland SN, Snowdon VK, Cappon A, Gordon-Walker TT, Williams MJ, Dunbar DR, Manning JR (2012). Differential Ly-6C expression identifies the recruited macrophage phenotype, which orchestrates the regression of murine liver fibrosis. Proc Natl Acad Sci USA.

[11] Issa R, Zhou X, Constandinou CM, Fallowfield J, Millward-Sadler H, Gaca MD, Sands E, Suliman I, Trim N, Knorr A, Arthur MJ, Benyon RC, Iredale JP (2004). Spontaneous recovery from micronodular cirrhosis: evidence for incomplete resolution associated with matrix cross-linking. Gastroenterology.

[12] Kim IH, Kisseleva T, Brenner DA (2015). Aging and liver disease. Curr Opin Gastroenterol.

[13] Pais R, Charlotte F, Fedchuk L, Bedossa P, Lebray P, Poynard T, Ratziu V, LIDO Study Group (2013). A systematic review of follow-up biopsies reveals disease progression in patients with non-alcoholic fatty liver. J Hepatol.

[14] Poynard T, Bedossa P, Opolon P (1997). Natural history of liver fibrosis progression in patients with chronic hepatitis C. The OBSVIRC, METAVIR, CLINIVIR, and DOSVIRC groups. Lancet.

[15] Wali M, Harrison RF, Gow PJ, Mutimer D (2002). Advancing donor liver age and rapid fibrosis progression following transplantation for hepatitis C. Gut.

[16] Tajiri K, Shimizu Y (2013). Liver physiology and liver diseases in the elderly. World J Gastroenterol.

[17] Collins BH, Holzknecht ZE, Lynn KA, Sempowski GD, Smith CC, Liu S, Parker W, Rockey DC (2013). Association of age-dependent liver injury and fibrosis with immune cell populations. Liver Int.

[18] Mahrouf-Yorgov M, Collin de l'Hortet A, Cosson C, Slama A, Abdoun E, Guidotti JE, Fromenty B, Mitchell C, Gilgenkrantz H (2011). Increased susceptibility to liver fibrosis with age is correlated with an altered inflammatory response. Rejuvenation Res.

[19] Gagliano N, Arosio B, Grizzi F, Masson S, Tagliabue J, Dioguardi N, Vergani C, Annoni G (2002). Reduced collagenolytic activity of matrix metalloproteinases and development of liver fibrosis in the aging rat. Mech Ageing Dev.

[20] Karsdal MA, Genovese F, Madsen EA, Manon-Jensen T, Schuppan D (2016). Collagen and tissue turnover as a function of age: implications for fibrosis. J Hepatol.

[21] Starkel P, Leclercq IA (2011). Animal models for the study of hepatic fibrosis. Best Pract Res Clin Gastroenterol.

[22] Watanabe T, Niioka M, Hozawa S, Kameyama K, Hayashi T, Arai M, Ishikawa A, Maruyama K, Okazaki I (2000). Gene expression of interstitial collagenase in both progressive and recovery phase of rat liver fibrosis induced by carbon tetrachloride. J Hepatol.

[23] Yang L, Kwon J, Popov Y, Gajdos GB, Ordog T, Brekken RA, Mukhopadhyay D, Schuppan D, Bi Y, Simonetto D, Shah VH (2014). Vascular endothelial growth factor promotes fibrosis resolution and repair in mice. Gastroenterology.

[24] Karlmark KR, Weiskirchen R, Zimmermann HW, Gassler N, Ginhoux F, Weber C, Merad M, Luedde T, Trautwein C, Tacke F (2009). Hepatic recruitment of the inflammatory Gr1+ monocyte subset upon liver injury promotes hepatic fibrosis. Hepatology.

[25] Adhyatmika A, Putri KS, Beljaars L, Melgert BN (2015). The Elusive Antifibrotic Macrophage. Front Med (Lausanne).

[26] Grenard P, Bresson-Hadni S, El Alaoui S, Chevallier M, Vuitton DA, Ricard-Blum S (2001). Transglutaminase-mediated cross-linking is involved in the stabilization of extracellular matrix in human liver fibrosis. J Hepatol.

[27] Kesteloot F, Desmoulière A, Leclercq I, Thiry M, Arrese JE, Prockop DJ, Lapière CM, Nusgens BV, Colige A (2007). ADAM metallopeptidase with thrombospondin type 1 motif 2 inactivation reduces the extent and stability of carbon tetrachloride-induced hepatic fibrosis in mice. Hepatology.

[28] Liu SB, Ikenaga N, Peng ZW, Sverdlov DY, Greenstein A, Smith V, Schuppan D, Popov Y (2016). Lysyl oxidase activity contributes to collagen stabilization during liver fibrosis progression and limits spontaneous fibrosis reversal in mice. FASEB J.

[29] Barry-Hamilton V, Spangler R, Marshall D, McCauley S, Rodriguez HM, Oyasu M, Mikels A, Vaysberg M, Ghermazien H, Wai C, Garcia CA, Velayo AC, Jorgensen B (2010). Allosteric inhibition of lysyl oxidase-like-2 impedes the development of a pathologic microenvironment. Nat Med.

[30] Delire B, Stärkel P, Leclercq I (2015). Animal Models for Fibrotic Liver Diseases: What We Have, What We Need, and What Is under Development. J Clin Transl Hepatol.

[31] Rikans LE, Kosanke SD (1984). Effect of aging on liver glutathione levels and hepatocellular injury from carbon tetrachloride, allyl alcohol or galactosamine. Drug Chem Toxicol.

[32] Bonnans C, Chou J, Werb Z (2014). Remodelling the extracellular matrix in development and disease. Nat Rev Mol Cell Biol.

[33] Iredale JP, Thompson A, Henderson NC (2013). Extracellular matrix degradation in liver fibrosis: biochemistry and regulation. Biochim Biophys Acta.

[34] Endo H, Niioka M, Sugioka Y, Itoh J, Kameyama K, Okazaki I, Ala-Aho R, Kähäri VM, Watanabe T (2011). Matrix metalloproteinase-13 promotes recovery from experimental liver cirrhosis in rats. Pathobiology.

[35] Higashiyama R, Inagaki Y, Hong YY, Kushida M, Nakao S, Niioka M, Watanabe T, Okano H, Matsuzaki Y, Shiota G, Okazaki I (2007). Bone marrow-derived cells express matrix metalloproteinases and contribute to regression of liver fibrosis in mice. Hepatology.

[36] Pellicoro A, Ramachandran P, Iredale JP, Fallowfield JA (2014). Liver fibrosis and repair: immune regulation of wound healing in a solid organ. Nat Rev Immunol.

[37] Imamura M, Ogawa T, Sasaguri Y, Chayama K, Ueno H (2005). Suppression of macrophage infiltration inhibits activation of hepatic stellate cells and liver fibrogenesis in rats. Gastroenterology.

[38] Mitchell C, Couton D, Couty JP, Anson M, Crain AM, Bizet V, Rénia L, Pol S, Mallet V, Gilgenkrantz H (2009). Dual role of CCR2 in the constitution and the resolution of liver fibrosis in mice. Am J Pathol.

[39] Barnes MA, McMullen MR, Roychowdhury S, Madhun NZ, Niese K, Olman MA, Stavitsky AB, Bucala R, Nagy LE (2015). Macrophage migration inhibitory factor is required for recruitment of scar-associated macrophages during liver fibrosis. J Leukoc Biol.

[40] Sahin H, Borkham-Kamphorst E, Kuppe C, Zaldivar MM, Grouls C, Al-samman M, Nellen A, Schmitz P, Heinrichs D, Berres ML, Doleschel D, Scholten D, Weiskirchen R (2012). Chemokine Cxcl9 attenuates liver fibrosis-associated angiogenesis in mice. Hepatology.

[41] Wasmuth HE, Lammert F, Zaldivar MM, Weiskirchen R, Hellerbrand C, Scholten D, Berres ML, Zimmermann H, Streetz KL, Tacke F, Hillebrandt S, Schmitz P, Keppeler H (2009). Antifibrotic effects of CXCL9 and its receptor CXCR3 in livers of mice and humans. Gastroenterology.

[42] Karlmark KR, Zimmermann HW, Roderburg C, Gassler N, Wasmuth HE, Luedde T, Trautwein C, Tacke F (2010). The fractalkine receptor CX3CR1 protects against liver fibrosis by controlling differentiation and survival of infiltrating hepatic monocytes. Hepatology.

[43] Trautwein C, Friedman SL, Schuppan D, Pinzani M (2015). Hepatic fibrosis: concept to treatment. J Hepatol.

[44] Wynn TA (2004). Fibrotic disease and the T(H)1/T(H)2 paradigm. Nat Rev Immunol.

[45] Hilmer SN, Cogger VC, Le Couteur DG (2007). Basal activity of Kupffer cells increases with old age. J Gerontol A Biol Sci Med Sci.

[46] Yang X, Liang L, Zong C, Lai F, Zhu P, Liu Y, Jiang J, Yang Y, Gao L, Ye F, Zhao Q, Li R, Han Z, Wei L (2016). Kupffer cells-dependent inflammation in the injured liver increases recruitment of mesenchymal stem cells in aging mice. Oncotarget.

[47] Seidler S, Zimmermann HW, Bartneck M, Trautwein C, Tacke F (2010). Age-dependent alterations of monocyte subsets and monocyte-related chemokine pathways in healthy adults. BMC Immunol.

[48] Popov Y, Sverdlov DY, Sharma AK, Bhaskar KR, Li S, Freitag TL, Lee J, Dieterich W, Melino G, Schuppan D (2011). Tissue transglutaminase does not affect fibrotic matrix stability or regression of liver fibrosis in mice. Gastroenterology.

[49] Verma S, Tachtatzis P, Penrhyn-Lowe S, Scarpini C, Jurk D, Von Zglinicki T, Coleman N, Alexander GJ (2012). Sustained telomere length in hepatocytes and cholangiocytes with increasing age in normal liver. Hepatology.

[50] Warren A, Cogger VC, Fraser R, Deleve LD, McCuskey RS, Le Couteur DG (2011). The effects of old age on hepatic stellate cells. Curr Gerontol Geriatr Res.

[51] McHutchison J, Goodman Z, Patel K, Makhlouf H, Rodriguez-Torres M, Shiffman M, Rockey D, Husa P, Chuang WL, Levine R, Jonas M, Theodore D, Brigandi R, Farglitizar Study Investigators (2010). Farglitazar lacks antifibrotic activity in patients with chronic hepatitis C infection. Gastroenterology.

[52] Deguchi JO, Aikawa E, Libby P, Vachon JR, Inada M, Krane SM, Whittaker P, Aikawa M (2005). Matrix metalloproteinase-13/collagenase-3 deletion promotes collagen accumulation and organization in mouse atherosclerotic plaques. Circulation.

[53] Dayan D, Hiss Y, Hirshberg A, Bubis JJ, Wolman M (1989). Are the polarization colors of picrosirius red-stained collagen determined only by the diameter of the fibers?. Histochemistry.

[54] Lattouf R, Younes R, Lutomski D, Naaman N, Godeau G, Senni K, Changotade S (2014). Picrosirius red staining: a useful tool to appraise collagen networks in normal and pathological tissues. J Histochem Cytochem.

