# Supplementary material for: Aging enhances liver fibrotic response in mice through hampering extracellular matrix remodeling
Source: Aging (Albany NY). 2016 Dec 9;9(1):98–111. doi: 10.18632/aging.101124 (PMC5310658; doi:10.18632/aging.101124)
Supplement: Supplementary file 1 [file aging-09-0098-s001.pdf]

SUPPLEMENTARY MATERIAL

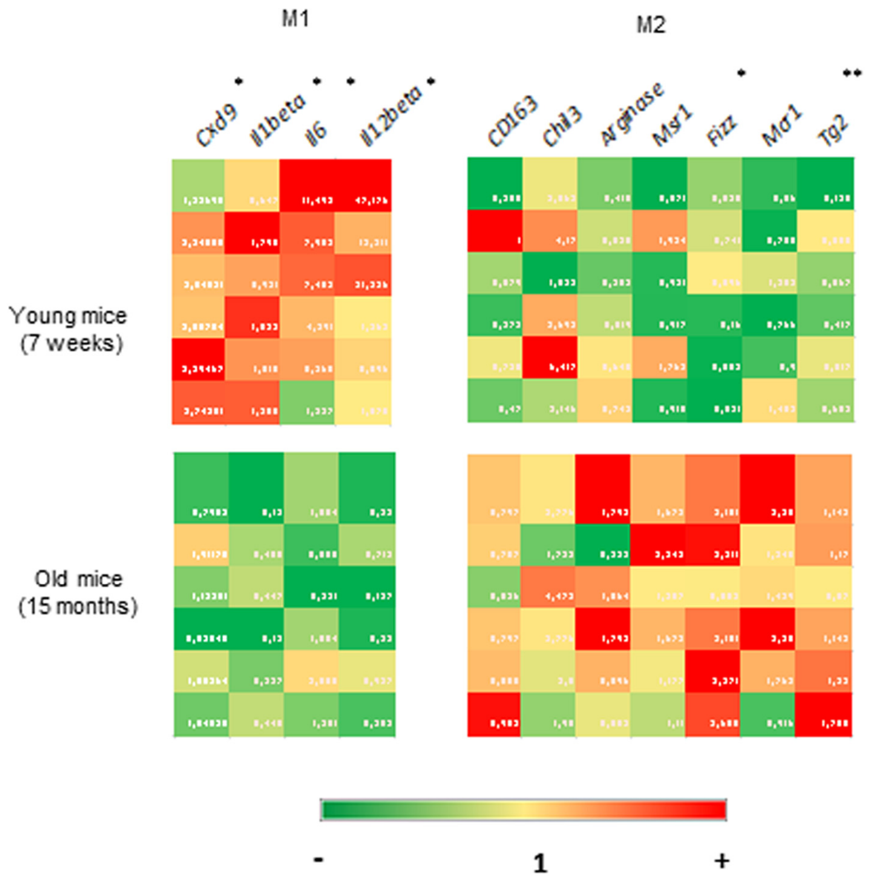

Supplementary Figure 1. Hepatic genes expression of M1 and M2 markers in young and old mice at peak of fibrosis. Induction compared to untreated liver appears in red and repression in green. Statistical analysis was performed by two-way ANOVA for repeated measures followed by Bonferroni's post-hoc correction. *P<0.05; **P<0.01 for differences between age groups.

Supplementary Table 1. Sequences of quantitative RT-PCR primers.

Genes	Forward sequence primer	Reverse sequence primer
Collagen I	TTCACCTACAGCACGCTTGT	TCTTGGTGGTTTTGTATTCGATGA
alpha Sma	TCCTGACGCTGAAGTATCCGATA	GGTGCCAGATCTTTTCCATGTC
Tgf beta	CCTGCAAGA CCATCGACATG	GAGCCTTAGTTTGGACAGGATCTG
Mmp2	GGACCCCGGTTTCCCTAA	CAGGTTATCAGGGATGGCATTC
Mmp3	TGGAGCTTCTGCAGAAATA	ATCTTTTGCAAGGCCATAG
Mmp8	GTCCCAAGTGGACACACACT	TCACTTCAGCCCTTGACAGC
Mmp9	TGAATCATAGAGGAAGCCCATTACA	CGGAGTCCAGCGTTGCA
Mmp13	CCAGAACTTCCCAACCATGT	GTCTTCCCCGTGTTCTCAAA
Mmp14	AGTCAGGGTCACCCACAAAGA	TTTGGGCTTATCTGGGACAGA
Timp1	CGCCTACACCCCAGTCATG	TGCGGTTCTGGGACTTGTG
Timp2	CATTACCCTCTGTGACTTCATTGTG	CTGTGGTTCAGGCTCTTCTTCTG
Cxcl9	TCTTGGGCATCATCTTCCTGG	GAGGTCTTTGAGGGATTTGTAGTGG
CD11b	GGGTCATTCGCTACGTAATTGG	TGTTCACCAGCTGGCTTAGATG
Ccl2	CCACTCACCTGC TGCTACTCAT	CTGCTGGT GATCCTCTTGT
F4/80	GATGAATTCCCGTGTTGTTGGT	ACATCAGTGTTCCAGGAGACACA
Vegf	TTACTGCTGTACCTCCACC	ACAGGACGGCTTGAAGATG
Mif	GCCAGAGGGGTTTCTGTCG	GTTCGTGCCGCTAAAAGTCA
Cx3cr1	ATCAGCATCGACCGGTACCT	CTGCACTGTCCGGTTGTTCAT
Il12beta	AGTGACATGTGGAATGGCGT	CAGTTCAATGGGCAGGGTCT
Il10	GATGCCCCAGGCAGAGAA	CACCCAGGGAATTCAAATGC
Il1beta	AGTTGACGGACCCCAAAAGA	GGACAGCCCAGGTCAAAGG
Il6	CTGCAA GAGACTTCCATCCAGTT	GAAG TAGGGAAGGC CGTGG
CD163	CAGGTGTTATCTGCTCCGAGTTC	CCATGTACCATTGTAAAACACTTCAA
Chil3	CATTGGAGGATGGAAGTTTGGA	GAATATCTGACGGTTCTGAGGAGTAGA
Arginase	TGGGTGGATGCTCACACTGA	CAGGTTGCCCATGCAGATT
Msr1	GAGGATGCTGACTGCAGTTCAG	GGGCCATTTTTAGTGCTGTGA
Mcr1	GCATGGGTTTTACTGCTACTTGATT	CAGGAATGCTTGTTCATATCTGTCTT
Fizz	ACTGCCTGTGCTTACTCGTTGA	TCCACCTCTTCATTCTTAGGACAGTT
Tg2	CCACGACCAGAACAGCAACC	GTATGTCCCTTCGCTCTTCTCC
Lox	CTACATCCAGGCTTCCAGG	GCGGAAATCGTAGCAGTA
Loxl2	GCTATGTAGAGGCCAAGTCCTG	CAGTGACACCCCAGCCATTG
Adamts2	CAGCCGCTACCTGCATTCCTATGA	CAGGCGCACACATAGTACCATCCA
